# Knowledge-Assisted Actor Critic Proximal Policy Optimization-Based Service Function Chain Reconfiguration Algorithm for 6G IoT Scenario

**DOI:** 10.3390/e26100820

**Published:** 2024-09-25

**Authors:** Bei Liu, Shuting Long, Xin Su

**Affiliations:** 1 School of Communication and Information Engineering, Chongqing University of Posts and Telecommunications, Chongqing 400065, China; 2 Department of Electronic Engineering, Tsinghua University, Beijing 100084, China

**Keywords:** 6G IoT, SFC reconfiguration, knowledge assisted, ACPPO algorithm

## Abstract

Future 6G networks will inherit and develop Network Function Virtualization (NFV) architecture. With the NFV-enabled network architecture, it becomes possible to establish different virtual networks within the same infrastructure, create different Virtual Network Functions (VNFs) in different virtual networks, and form Service Function Chains (SFCs) that meet different service requirements through the orderly combination of VNFs. These SFCs can be deployed to physical entities as needed to provide network functions that support different services. To meet the highly dynamic service requirements in the future 6G Internet of Things (IoT) scenario, the highly flexible and efficient SFC reconfiguration algorithm is the key research direction. Deep-learning-based algorithms have shown their advantages in solving this type of dynamic optimization problem. Considering that the efficiency of the traditional Actor Critic (AC) algorithm is limited, the policy does not directly participate in the value function update. In this paper, we use the Proximal Policy Optimization (PPO) clip function to restrict the difference between the new policy and the old policy, to ensure the stability of the updating process. We combine PPO with AC, and further bring the historical decision information as the network knowledge to offer better initial policies, to accelerate the training speed. We also propose the Knowledge = Assisted Actor Critic Proximal Policy Optimization (KA-ACPPO)-based SFC reconfiguration algorithm to ensure the Quality of Service (QoS) of end-to-end services. Simulation results show that the proposed KA-ACPPO algorithm can effectively reduce computing cost and power consumption.

## 1. Introduction

Looking towards 2030 and beyond, 6G will provide lower latency and more reliable end-to-end connectivity to power Industry 4.0 to Industry 5.0 [1]. The network environment and service requirements are highly dynamic in future 6G IoT scenarios. To cope with highly dynamic service requirements, 6G networks will inherit and develop NFV architecture. With the NFV-enabled network, it becomes possible to establish different virtual networks within the same infrastructure and create different VNFs in different virtual networks. When the service requirements arrive, VNFs are combined in an orderly manner to form SFCs to meet different service requirements. In this way, SFC deployment based on mobile edge computing and network function virtualization can be a viable solution for providing flexible and controlled network services [2]. When the service requirement changes, the corresponding SFC needs to be reconfigured to guarantee the QoS requirements. To meet the low latency requirements of 6G services, highly flexible and efficient SFC orchestration and reconfiguration algorithms have become a crucial problem.

The SFC reconfiguration problem involves the study of how to modify, re-organize, and monitor the deployed SFC to cope with network dynamic changes and meet new service requirements. SFC reconfiguration is always implemented by VNF migration. Reference [3] modeled the SFC reconfiguration problem as an Integer Linear Programming (ILP) formulation and proposed a greedy-based heuristic algorithm to solve the problem Reference [4] proposed a threshold-dependent scalable cluster VNF migration algorithm to minimize the cost of embedding VNF under the condition of satisfying delay constraints. Reference [5] described the VNF migration problem as a new graph theory problem and proposed an efficient heuristic algorithm based on dynamic programming to effectively alleviate dynamic traffic and reduce total traffic costs. Reference [6] studied the reconfiguration problem of a set of SFCs with different priorities. A polynomial time heuristic algorithm was proposed to quickly deploy emergency SFCs while meeting the requirements of emergency SFC input traffic and resource constraints. Another precise algorithm was also proposed to achieve maximum profit for service providers. Reference [7] aimed to minimize the total migration cost and used a temporal conventional network to predict network traffic, proposing a fast and efficient heuristic VNF migration algorithm.

Nowadays, with the rapid development of Artificial Intelligence (AI), deep learning has been widely investigated and has shown advantages in coping with this type of dynamic problem. Reference [8] proposed a DRL-based algorithm to provide fast VNF migration decisions in highly dynamic environments, with the goal of minimizing the weighted total latency and cost of VNF migration. Reference [9] used a mixed-density neural network to accurately model complex user migration patterns in reality, in order to support the prediction of user edge cloud access probabilities and minimize the sum of operational costs and potential losses caused by downtime. Reference [10] proposed a deep Dyna-Q approach to solve the SFC reconfiguration problem under the premise of guaranteeing QoS and resource constraints. Reference [11] proposed a SFC management scheme based on the prediction of service. Reference [12] proposed the Dueling Double Deep Q Network (D3QN)-based SFC orchestration scheme to ensure QoE of users in the highly dynamic and resource-constrained Unmanned Aerial Vehicle (UAV) scenario.

The aforementioned research demonstrates the tremendous potential of deep learning algorithms. The AC algorithm, as a policy-based algorithm, has been used in many dynamic optimization problems. However, the efficiency of classical AC is limited in that the policy does not directly participate in the value function update. So, we use the principle of PPO, that is, proximal ratio pruning, to limit the magnitude of policy updates, and form the ACPPO algorithm. Based on this, we further utilize the historical decision information as the network knowledge to offer better initial policies and accelerate the training speed.

The main contributions of this paper are synthesized as follows:

- We formulate the SFC reconfiguration as a VNF migration problem, aiming to minimize the migration cost, to cope with the highly dynamic service requirements in 6G IoT scenarios.
- We use proximal ratio pruning to limit the magnitude of policy updates, reducing the impact of coupling relationship in the AC algorithm, and combine the advantages of the AC and PPO algorithm to solve the VNF migration problem. And we further introduce the 6G knowledge base, which is dynamic updating by saving and updating the excellent historical policies, to expand the action space and formulate the KA-ACPPO algorithm.
- Simulation results show that our proposed KA-ACPPO can effectively reduce computing cost and power consumption.

This paper is organized as follows. In Section 1, the system model is presented and the problem is formulated. Section 2 proposes the KA-ACPPO algorithm. Section 3 gives the simulation results. Finally, Section 4 concludes the paper and gives future research directions.

## 2. System Model and Problem Formulation

### 2.1. Network Model

Consider the 6G IoT scenario. Service requirements are satisfied by SFC orchestration. SFC orchestration is complemented by global control with the help of the 6G knowledge base. When the device produces a service request, it is assigned to the closed-edge node and communicates with the top-level SDN controller and NFV manager. The VNF manager complements the VNF orchestration, forming an ordered set of VNFs, that is, the SFC deployed policy. Finally, the policy is issued to the infrastructure layer to deploy the network functions to meet the service requirements. And when the service requirements change, SFC can be reconfigured through VNF migration, for example, VNF is migrated from one edge node to another edge node.

**Figure 1:**
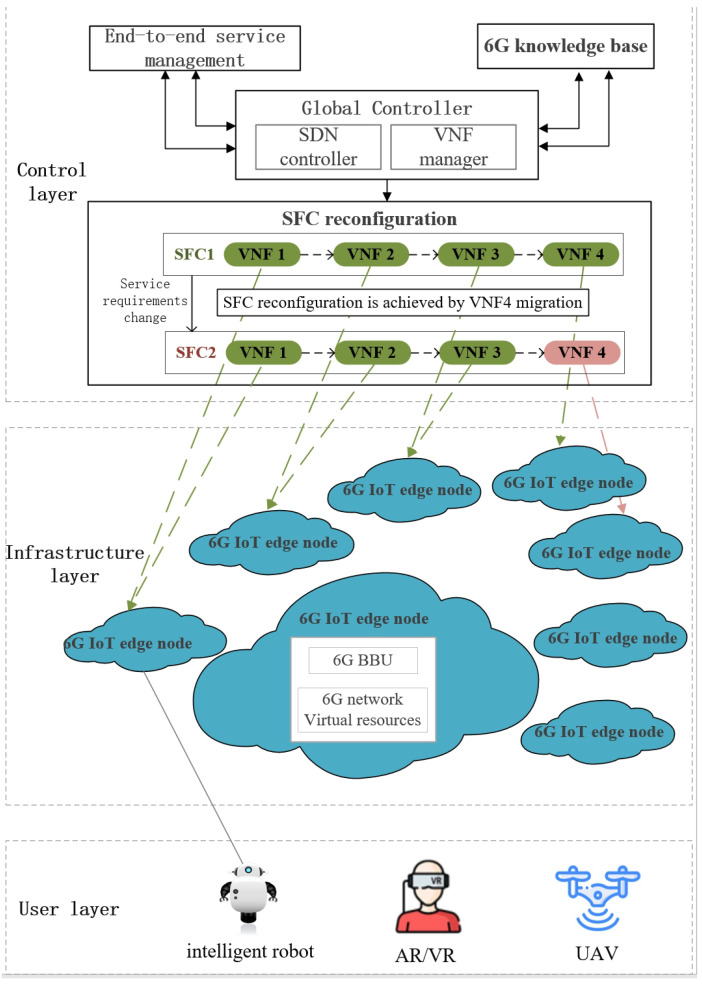
6G IoT scenario and system model.

It is shown in Figure 1 that the edge nodes and the physical links in the infrastructure layer are expressed as the undirected graph G=(X,L), in which *X* and *L*, respectively, describe the set of the edge nodes and the physical links. The available computing resource of the edge node x∈X can be expressed as $C_{x}^{CPU}$. $l_{x,y}$ describes the physical link between the edge node *x* and the edge node *y*, in which x,y∈X and x≠y. SFC reconfiguration is implemented through VNF migration, so the physical link needs to reserve bandwidth *B* for VNF migration. In fact, every SFC can be expressed as an ordered set of VNF, denoted as $V=\{v_{1},v_{2},\cdots ,v_{\left|V\right|}\}$. We divide IoT services into two categories: delay-sensitive services and delay-tolerant services. The corresponding SFC requests can be denoted as delay-sensitive request j∈J and delay-tolerant request k∈K, and the corresponding computing resource provided by the edge nodes can be, respectively, denoted as $b_{j}$ and $b_{k}$. This paper ensures the QoS of service requests by controlling the reconstruction costs of VNF migration in three aspects: computing cost, power consumption cost, and delay cost.

For clear presentation, we summarize the notations used in the following formulation in Table 1.

### 2.2. The Computing Cost of VNF Migration

SFCs are deployed in the edge node and are allocated computing resources from the edge node to meet the service requirements. To represent the computing resource consumption of node *x*, we denote the decision-making variables of SFC reconfiguration as follows:

(1) $$\theta_{x,j}=\left\{\begin{array}{cc} 1 & if\;resource\;b_{j}\;is\;allocates\;to\;request\;j\\ 0 & otherwise \end{array}\right.,$$

(2) $$\theta_{x,k}=\left\{\begin{array}{cc} 1 & if\;resource\;b_{k}\;is\;allocates\;to\;request\;k\\ 0 & otherwise \end{array}\right..$$

The computing resource consumption of the edge node *x* can be denoted as follows:

(3) $$z_{k}=\sum_{j=1}^{J}\theta_{x,j}b_{j}+\sum_{k=1}^{K}\theta_{x,k}b_{k}.$$

During the VNF migration process, only the CPU state between the initial node and the target node is considered. Therefore, the computing cost of VNF migration can be denoted as follows

(4) $$Cost_{1}=\sum_{x=1}^{X}\sum_{v=1}^{V}W_{x}^{V}\left(1-U_{x}^{V}\right)z_{k},$$

where $U_{x}^{V}$ is the variable of SFC deployment, and $W_{x}^{V}$ is the variable of SFC reconfiguration. For example, if the first VNF of the current SFC is not deployed at edge node 1, it can be denoted as $U_{1}^{1}=0$. If the VNF is migrated to the edge node 1, it can be denoted as $W_{1}^{1}=1$, and $W_{1}^{1,1}\left(1-U_{1}^{1,1}\right)=1$, which means the VNF is migrated.

### 2.3. The Power Consumption Cost of VNF Migration

VNF migration can cause changes in the working status of certain edge nodes, resulting in additional power consumption. Power consumption is related to the usage of computing resources at edge nodes. Based on the current SFC deployment policy, the power consumption of the edge node *x* can be denoted as [13]:

(5) $$P_{x}=P\left(0\%\right)+\left(P\left(100\%\right)-P\left(0\%\right)\right)\left(2z_{x}-{\left(z_{x}\right)}^{1.4}\right),$$

where P(0%) denotes the power consumption of the edge nodes in idle modes, and P(100%) denotes the power consumption of the edge nodes in full-load modes.

Therefore, the power consumption cost of VNF migration can be denoted as:

(6) $$Cost_{2}=\sum_{x=1}^{X}\sum_{v=1}^{V}W_{x}^{V}\left(1-U_{x}^{V}\right)P_{x}.$$

### 2.4. The Delay Cost of VNF Migration

In this paper, the delay-sensitive services and the delay-tolerate services are both modeled as M/M/1 queuing, where the arriving rates are, respectively, depicted as $\lambda_{j}$ and $\lambda_{k}$. And the total arriving rate at edge node *x* can be denoted as follows:

(7) $$\lambda^{\prime }=\sum_{j=1}^{J}\lambda_{j}+\sum_{k=1}^{K}\lambda_{k}.$$

The CPU working frequency of the edge node *x* is denoted as *F*, and the serving rate of VNF can be denoted as:

(8) $$\mu^{\prime }=z_{x}F.$$

And the traffic intensity can be denoted as:

(9) $$\rho^{\prime }=\frac{\lambda^{\prime }}{\mu^{\prime }}.$$

Therefore, the queuing delay of the SFC requests can be denoted as:

(10) $$D_{q}=\frac{{\rho^{\prime }}^{2}}{\lambda (1-\rho^{\prime })}.$$

Simply, only the queuing delay of the delay-sensitive SFC requests is considered, which is denoted as:

(11) $$D_{j}=\frac{\beta_{j}}{b_{j}},$$

where $\beta_{j}$ denotes the data size of the *j*th delay-sensitive SFC request.

Considering the network overhead of the infrastructure layer and the stability of network services, the impact of VNF migration latency and downtime should be minimized to the greatest extent possible. The VNF migration latency is related to the position change of VNF, and the number of migrated VNF at edge node *x* can be denoted as:

(12) $$\mu_{x}=\sum_{v=1}^{V}\left|W_{x}^{V}-U_{x}^{V}\right|.$$

And the number of VNF migration between edge node *x* and edge node *y* can be denoted as:

(13) $$\mu_{x,y}=\sum_{v=1}^{V}W_{x}^{V}\left(1-U_{y}^{V}\right).$$

The corresponding delay of VNF migration between edge node *x* and edge node *y* can be denoted as:

(14) $$D_{x,y}^{v}=\frac{d_{j}}{B}W_{x}^{v}U_{y}^{v}\max_{(x,y)\in X}\left(\mu_{x},\mu_{x,y},\mu_{y}\right).$$

The total delay of VNF migration caused by the position change of VNFs can be denoted as:

(15) $$D_{m}=\max_{\left(v,x,y\right)}D_{x,y}^{v}.$$

Downtime refers to the time period during which the migrated VNF is unresponsive, as the VNF state is either migrating from the initial node to the target node or the network has not yet aggregated. Therefore, it is necessary to minimize the possibility of network service interruption caused by prolonged downtime. Using the current resource utilization rate of network edge nodes as a penalty factor to calculate the additional delay caused by excessive downtime, the CPU utilization rate of current network edge node *x* can be expressed as:

(16) $$\rho_{x}=\frac{z_{x}}{C_{x}^{CPU}}.$$

The additional delay caused by prolonged downtime is denoted as

(17) $$D_{o}=\sum_{x=1}^{X}\sum_{v=1}^{V}\left(D_{j}+D_{q}\right)W_{x}^{v,s}\left(1-U_{x}^{v,s}\right)\rho_{x}.$$

When the downtime of VNF migration is too long, it can lead to delay-sensitive SFC requests not being processed in a timely manner during migration, resulting in QoS degradation of delay-sensitive SFC requests. The QoS degradation rate of delay-sensitive SFC requests caused by long VNF downtime can be expressed as:

(18) $$Q_{j}=\frac{D_{o}}{D_{q}+D_{j}+D_{m}+D_{o}}.$$

Taking into account the total latency of VNF migration and the additional latency caused by excessive downtime, the latency cost of VNF migration can be expressed as:

(19) $$Cost_{3}=D_{m}+D_{o}.$$

### 2.5. Problem Formulation

The total cost of SFC reconfiguration in 6G IoT scenario can be denoted as:

(20) $$Cost=\frac{Cost_{1}}{\sigma_{1}}+\frac{Cost_{2}}{\sigma_{2}}+\frac{Cost_{3}}{\sigma_{3}},$$

where $\sigma_{1}$, $\sigma_{2}$, and $\sigma_{3}$ are normalized constants. In this paper, we minimize the total cost of SFC reconfiguration, and formulate the problem as follows

(21) $$\min_{U_{x}^{V},W_{x}^{V}}\left(Cost=\frac{Cost_{1}}{\sigma_{1}}+\frac{Cost_{2}}{\sigma_{2}}+\frac{Cost_{3}}{\sigma_{3}}\right),$$

Satisfy the constraint:

(22) $$\begin{array}{c} \left\{\begin{array}{cc} \; & C_{1}:z_{x}\leq C_{x}^{CPU},\forall j\in X\\ \; & C_{2}:\sum_{x=1}^{X}W_{x}^{V}=v,\forall v\in V\\ \; & C_{3}:D_{q}+D_{j}+D_{m}+D_{o}\leq D,\forall v\in V,\forall a,y\in X\\ \; & C_{4}:z_{x}F-\left(\sum_{j=1}^{J}\lambda_{j}+\sum_{k=1}^{K}\lambda_{k}\right)\geq 0,\forall x\in X,\forall j\in J,\forall k\in K \end{array}\right., \end{array}$$

where $C_{1}$ ensures that the edge node *x* would not be overloaded. $C_{2}$ ensures that reasonable quantity of VNFs are deployed in every SFC. $C_{3}$ is the constraint of delay constraint of the delay-sensitive services. $C_{4}$ ensures the stability of the SFC request queueing.

## 3. Knowledge-Assisted ACPPO Algorithm

In this section, the Markov Decision Process (MDP) is used to model the above problem. The MDP can be simply defined as <S,A,R>, where state *S*, action *A*, and reward *R* are, respectively, defined as follows.

### 3.1. State

Define the state *S* as system state at time *t*, which can be written as follows:

(23) $$s\left(t\right)=\{c_{j}\left(t\right),b_{k}\left(t\right),z_{x}\left(t\right)\},$$

where $c_{j}\left(t\right)$ and $b_{k}\left(t\right)$, respectively, denote the resource requirements of delay-sensitive and delay-tolerate SFC requests, which are detailed as follows:

(24) $$c_{j}\left(t\right)=\left\{c_{1}\left(t\right),c_{2}\left(t\right),\cdots ,c_{J}\left(t\right)\right\},$$

(25) $$b_{k}\left(t\right)=\left\{b_{1}\left(t\right),b_{2}\left(t\right),\cdots ,b_{K}\left(t\right)\right\},$$

(26) $$z_{x}\left(t\right)=\left\{z_{1}\left(t\right),z_{2}\left(t\right),\cdots ,z_{X}\left(t\right)\right\}.$$

### 3.2. Action

Action *A* denotes the sets of VNF migration, and the VNF migration at time *t* can be denoted as

(27) $$a\left(t\right)=\{a_{1}\left(t\right),a_{2}\left(t\right),\cdots ,a_{i}\left(t\right),\cdots ,a_{V}\left(t\right)\},\forall i\in V,$$

where $a_{i}\left(t\right)$ describes that if the *i*th VNF is migrated. $a_{i}\left(t\right)=1$ means that *i*th VNF is migrated, and otherwise, $a_{i}\left(t\right)=0$. In order to better adapt to the network changes, the knowledge-molded VNF transfer method is introduced into the 6G network knowledge base as prior knowledge to expand the action space, allowing for more choices in VNF transfer decision-making and optimization processes, thereby improving the flexibility and adaptability of the algorithm. At the same time, it is possible to explore more fully, thus having the opportunity to find better solutions and improve algorithm performance and efficiency.

### 3.3. Reward

Since the section aims to minimize the configuration cost of SFC, the reward function can be denoted as

(28) r(t)=−αCost,

where α>0 is the reward factor, and the larger r(t), the smaller SFC reconfiguration cost.

Based on the above MDP model, the KA-ACPPO algorithm is proposed to solve the SFC reconfiguration problem. Combining the advantages of the AC algorithm and the PPO algorithm, the ACPPO algorithm is efficient, stable, and adaptable. However, the selection of hyperparameters and the efficiency of sample processing are still issues that need to be considered when using the ACPPO algorithm. Therefore, under the 6G autonomous control framework, the KA-ACPPO algorithm introduces network knowledge related to the algorithm from the 6G network knowledge base, initializes the Actor network parameters and the Critical network parameters, and accelerates the learning process and reduces training time by providing a good initial strategy in the initial stage, avoiding a lot of exploration. Meanwhile, another VNF transfer method is introduced from the 6G network knowledge base as prior knowledge to expand the action space, helping the algorithm converge and learn faster, improving its performance in complex tasks, and enabling it to cope with a wider range of 6G scenarios.

**Figure 2:**
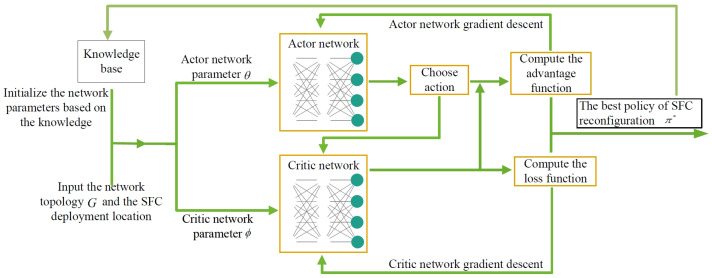
The framework of the proposed KA-ACPPO algorithm.

The framework of the KA-ACPPO algorithm is shown in Figure 2. Input the current network topology *G* and the VNF deployment state, then the Actor network factor and the Critic network factor are initialized according to the module of the 6G knowledge base. The historic policies in the module knowledge base are updated based on the SFC reconfiguration policy. The Actor network is responsible for selecting VNF migration actions, while the Critic network evaluates the value of each action. The gradient descent method is used to update the parameters of the Critic network and the Actor network, and the optimal SFC reconstruction strategy can be denoted as:

(29) $$\pi^{*}=\arg\max_{a}V^{\pi }\left(s,a\right),\forall s,a.$$

The Critical network evaluates the Actor network by using the advantage function to select actions based on the current output strategy, thereby assisting the Actor in policy updates. In the current state s(t), the advantage function of selecting action a(t) based on the strategy of the Actor network can be denoted as:

(30) $${\hat{A}}_{t}=Q_{\pi old}\left(s_{t},a_{t}\right)-V_{\pi old}\left(s_{t}\right),$$

where $Q_{\pi old}\left(s_{t},a_{t}\right)$ denotes the value $V_{\pi old}\left(s_{t}\right)$ is the value at state $s_{t}$.

According to generalized advantage estimation [14], the advantage function can be denoted as:

(31) $${\hat{A}}_{t}=\theta_{t}+\left(\gamma \lambda \right)\theta_{\left(t+1\right)}+\cdots +{\left(\gamma \lambda \right)}^{T-t+1}\theta_{\left(T-1\right)},$$

where $\theta_{t}=r\left(t\right)+\gamma V_{\left(s_{t+}\right)}-V_{\left(s_{t}\right)}$ is the error of Temporal Difference (TD), and γ is the discount factor, Let λ=1, the advantage function can be denoted as:

(32) $${\hat{A}}_{t}=-V\left(s_{t}\right)+r_{t}+\gamma r_{t+1}+\cdots +\gamma^{T-t+1}r_{t-1}+\gamma^{T-t}V\left(s_{T}\right).$$

The larger the value of the advantage function, the better the selected action. The advantage function is designed to increase the stability of policy and avoid unstable optimization processes caused by excessive policy updates. At the same time, by limiting the amplitude of action policy updates, the Actor network’s action update amplitude will not be too large. The update amplitude of the Actor network action strategy represents the ratio of the probability of the current strategy taking action a(t) in state s(t) to the probability of the old strategy taking action a(t) in state s(t), which is denoted as:

(33) $$r_{t}\left(\theta \right)=\frac{\pi_{\theta }\left(a_{t}|s_{t}\right)}{\pi_{\theta_{old}}\left(a_{t}|s_{t}\right)}.$$

The clip function limits the size of the policy update step, preventing excessive policy updates and thus avoiding instability issues. The clip function can be denoted as:

(34) $$clip\left(r_{t}\left(\theta \right),1-\varepsilon ,1+\varepsilon \right)=\left\{\begin{array}{cc} 1+\varepsilon & r_{t}\left(\theta \right)>1+\varepsilon \\ 1-\varepsilon & r_{t}\left(\theta \right)>1+\varepsilon \\ r_{t}\left(\theta \right) & otherwise \end{array}\right..$$

The clip function restrict the difference between the new policy and the old policy, to ensure the stability of the updating process. The proximal clip loss function in this section can be denoted as:

(35) $$L^{clip}\left(\theta \right)=E\left[\min r_{t}\left(\theta \right){\hat{A}}_{t},clip\left(r_{t}\left(\theta \right),1-\varepsilon ,1+\varepsilon \right){\hat{A}}_{t}\right].$$

Critic network updates as follows:

(36) $$\phi =\phi +\beta \nabla_{\phi }L\left(\phi \right).$$

where β is the learning rate of the Critic network, and L(ϕ) is the loss function of the Critic network to minimize TD error, which can be denoted as:

(37) $$L\left(\phi \right)=r_{t}+\gamma r_{t+1}+\cdots +\gamma^{T-t+1}r_{T-1}+\gamma^{T-1}V{\left(s\left(T+1\right)-V\left(s\left(T\right)\right)\right)}^{2}.$$

The Actor network updates as follows:

(38) $$\theta =\theta +\beta^{\prime }\nabla_{\theta }L^{clip}\left(\theta \right),$$

where $\beta^{\prime }$ is the learning rate of the Actor network. Algorithm 1 detailed the training process of KA-ACPPO.
**Algorithm 1** The Training Process of KA-ACPPO.1:**Input**: the current network topology *G* and current VNF deployment location2:**Output**: the best SFC reconfiguration policy $\pi^{*}$3:Initialize the Actor network factor θ and the Critic network factor ϕ4:**for** thread−number=1,⋯,N **do**5:    **for** interation−number=1,⋯,K **do**6:        Obtain the current network state s(t), and bring into the knowledge-based VNF migration to expand the action space7:        Select the action a(t) according to the policy of Actor network8:        **if** the constraints $C_{1}\;C_{4}$ are satisfied **then**9:           Execute the action a(t), get the instant reward r(t), and turn to the next state s(t+1)10:        **else**11:           Reselect the action a(t)12:        **end if**13:    **end for**14:    Calculate ${\hat{A}}_{t}$ and $L^{clip}\left(\theta \right)$15:    Update the Critic network based on Equation (35)16:    Update the Actor network based on Equation (37)17:**end for**

## 4. Simulation and Result Analysis

### 4.1. Parameter Setting

In this section, we set up the simulation environment to evaluate the performance of the proposed KA-ACPPO algorithm. The key parameters are listed in Table 2 [15]. And it is worth noting that the parameter setting of the service requests are generated by random selection from the sum of Gaussian functions with different parameters [15].

### 4.2. Results Analysis

**Figure 3:**
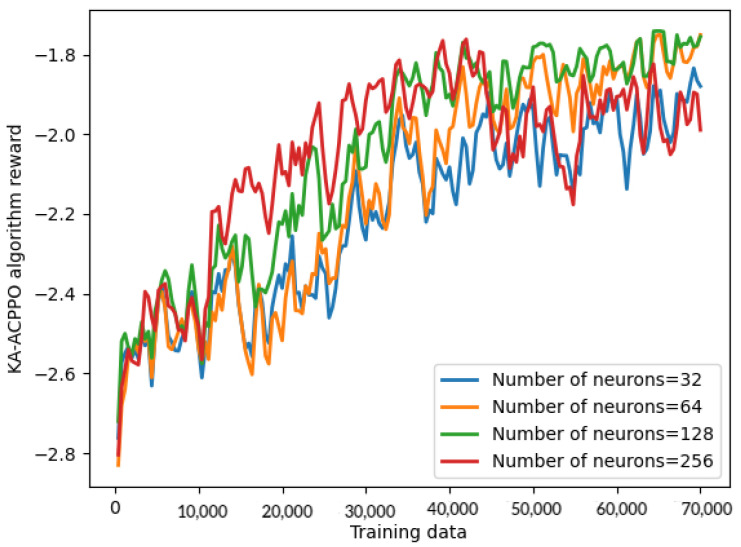
Rewards of KA-ACPPO algorithm for different number of neurons.

Figure 3 shows KA-ACPPO algorithm rewards for different number of neurons. It can be seen that, as the number of neurons increases, the reward of the KA-ACPPO algorithm also increases. The reason is that increasing the number of neurons can improve the representation ability of neural networks, enabling them to better learn and fit complex functional relationships, and enable the network to better express the complex relationships between states and actions, thereby improving rewards. However, increasing the number of hidden layer neurons can increase the complexity of the model and may lead to a slower training process. Therefore, when selecting the number of hidden layer neurons, it is necessary to balance training efficiency and increasing rewards. Considering training efficiency and rewards comprehensively, the number of neurons for the KA-ACPPO algorithm is determined to be 128 in the following simulation in this paper.

**Figure 4:**
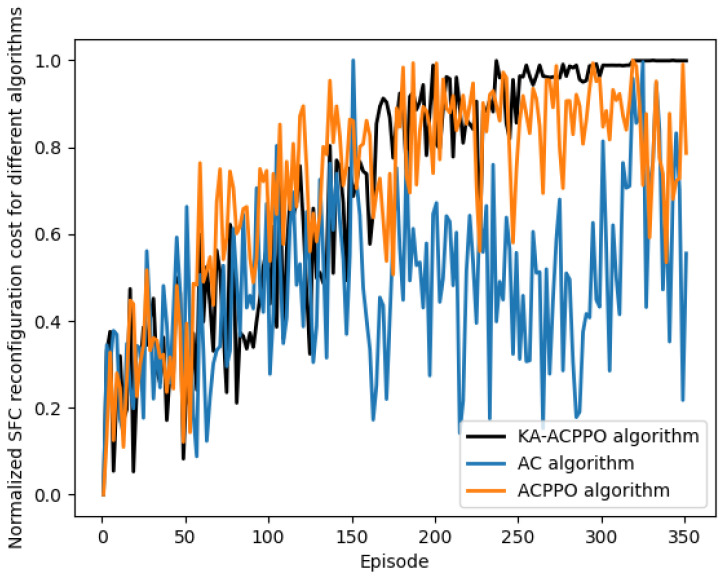
The normalized reconfiguration cots for different algorithm.

Figure 4 shows the normalized reconfiguration costs for different algorithms, which is our optimization object in Equation (21). From the figure, we can see that the AC algorithm can quickly converge into a higher convergence value, but it fluctuates greatly throughout the entire training cycle, resulting in a lower convergence value. This is because the strategies and value functions in the AC algorithm are interdependent, and their updates affect each other. This coupling relationship leads to fluctuations in the training process of the AC algorithm. ACPPO algorithm uses proximal ratio pruning to limit the magnitude of policy updates, reducing the impact of this coupling relationship to ensure a stable training process. Moreover, the proposed KA-ACPPO algorithm introduces 6G knowledge on the basis of the ACPPO algorithm, which helps the algorithm learn and converge quickly, ultimately achieving smaller fluctuations and higher convergence.

**Figure 5:**
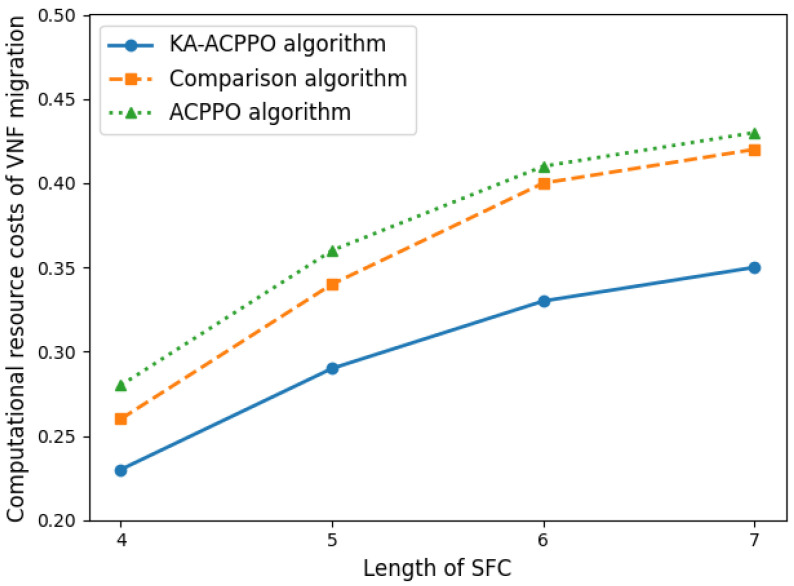
The computing cost of VNF migration for different algorithms.

Figure 5 shows the computing cost for VNF migration using different algorithms, which is related to Equation (4). The length of SFC affects the computational resource cost of VNF migration from two aspects: the current usage of computing resources of edge nodes, and the number of VNF migrations. As the length of SFC increases, the number of VNFs per SFC increases, and the current usage of edge node computing resources becomes more complex. With the goal of minimizing reconstruction costs, the number of VNF migrations should be controlled while migrating VNFs that use fewer computing resources. From the figure, it can be also observed that as the length of SFC increases, the computational resource cost of VNF migration for all three algorithms increases. The computational resource cost of VNF migration using the proposed KA-ACPPO algorithm is always lower than the other two algorithms, ultimately controlling the computational resource cost of VNF migration at 34%.

**Figure 6:**
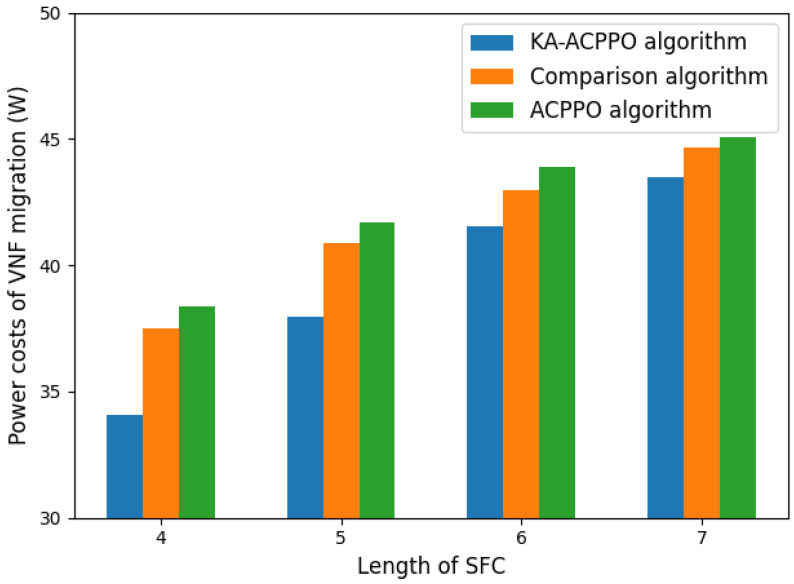
The power consumption cost of VNF migration for different algorithms.

Figure 6 shows the power costs of VNF migration for different algorithms, which is related to Equation (6), and the comparison algorithm is Reinforcement Learning (RL)-based VNF deployment scheme proposed in [16]. The power cost of VNF migration is related to the usage of network node computing resources. Therefore, the length of SFC also affects the power cost of VNF migration in two aspects: the current usage of edge node computing resources and the number of VNF migrations. As the length of SFC increases, the number of VNFs per SFC increases, and the computing resources of the network edge nodes currently used increase, The power consumption increases accordingly. Considering that VNF migration may change the working mode of edge nodes, with the goal of minimizing reconstruction costs, the number of VNF migrations should be controlled to minimize the additional power consumption caused by such changes. From Figure 6, it can be observed that as the length of SFC increases, the power cost of VNF migration for all three algorithms increases. The power cost of VNF migration for the KA-ACPPO algorithm is always lower than the other two algorithms.

**Figure 7:**
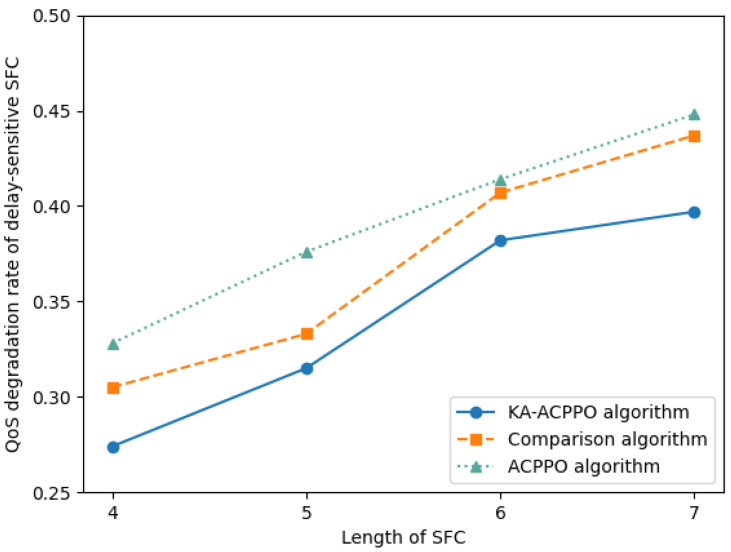
QoS degradation rate of delay-sensitive SFC for different algorithms.

Figure 7 shows the QoS degradation rates, which is related to Equation (18), of delay-sensitive SFC requests using different algorithms. According to Formula (18), the QoS degradation rate of delay-sensitive SFC is defined as the proportion of additional delay caused by excessive downtime to the total end-to-end delay. The length of SFC affects the QoS degradation rate of delay-sensitive SFCs from two aspects. Firstly, as the length of the SFC increases, the total end-to-end latency also increases. Secondly, as the length of the SFC increases, the number of VNF migrations per SFC may also increase. The negative impact of migration latency and downtime on the QoS of delay-sensitive SFC will also increase. From Figure 7, it can be observed that as the length of the SFC increases, the QoS degradation rate of the delay-sensitive SFC of all three algorithms increases. The QoS degradation rate of the delay-sensitive SFC of the KA-ACPPO algorithm is always lower than the other two algorithms, and the QoS degradation rate of the delay-sensitive SFC is ultimately controlled at 39%.

## 5. Conclusions and Discussion

6G will inherit and develop NFV architecture. With this SFC-enabled flexible network architecture, SFC orchestrates different VNFs to improve maintaining the QoS of different services. Considering that the coupling relationship leads to fluctuations in the training process of the traditional AC algorithm, this paper brings proximal ratio pruning to limit the magnitude of policy updates and combine the advantages of the AC algorithm and the PPO algorithm. Moreover, we propose to introduce the 6G knowledge base to provide a better initial policy to accelerate training speed and form the KA-ACPPO-based VNF migration algorithm. Simulation results show that the proposed algorithm can effectively reduce computing cost and power consumption.

However, in this paper, we consider using historic policies as network knowledge to improve performance. In future work, more dimensions of knowledge, such as model-based algorithms and expert experiences, could be researched to further improve the performance of deep learning algorithms.

## Figures and Tables

**Table 1:** Notations used in this paper.

Notation	Description
*X*	the set of edge nodes
*L*	the set of physical links
$l_{xy}$	the link between edge node *x* and edge node *y*
j∈J	the index of delay-sensitive requests
k∈K	the index of delay-tolerant requests
$b_{j}$	computing resource allocated to request *j*
$b_{k}$	computing resource allocated to request *k*
$z_{x}$	the computing resource consumption of edge node *x*
$U_{x}^{V}$	the variable of SFC deployment
$W_{x}^{V}$	the variable of SFC reconfiguration
$P_{x}$	the power consumption cost
$\lambda_{j},\lambda_{k}$	the arriving rates of delay-sensitive and delay-tolerant requests
$\lambda^{\prime }$	total arriving rate of requests
$\mu^{\prime }$	the serving rate of VNF
$\rho^{\prime }$	the traffic intensity
$D_{q}$	the queuing delay of SFC requests
$D_{j}$	the delay of the delay-sensitive SFC requests
$\beta_{j}$	the data size of *j*th delay-sensitive SFC requests
$D_{m}$	the total delay of VNF migration caused by the position change of VNFs
$D_{o}$	the additional delay caused by prolonged downtime
$Q_{j}$	QoS degradation rate of *j*th delay-sensitive SFC requests

**Table 2:** Simulation parameters.

Simulation Parameters	Simulation Values
number of edge nodes	10
computation resource of edge nodes	[100,200] (units)
power consumption of the edge node at idle state	87 (W)
power consumption of the edge node at full-load state	145 (W)
reserve bandwidth for VNF	[5,15] (Mbps)
required computing resource for delay-sensitive SFC	[10,30] (units)
required computing resource for delay-tolerate SFC	[5,15] (units)
length of SFC	[4,5,6,7]
end-to-end delay constraint	60 (ms)
the parameter of clip function	0.2
learning rate of Actor network	0.001
learning rate of Critic network	0.01
discount factor	0.99

## Data Availability

No new data were created.

## Abbreviations

The following abbreviations are used in this manuscript:

NFV	Network Function Virtualization
VNF	Virtual Network Function
SFC	Service Function Chains
IoT	Internet of Things
AC	Actor Critic
PPO	Proximal Policy Optimization
QoS	Quality of Service
AI	Artificial Intelligence
DQN	Deep Q Network
D3QN	Dueling Double Deep Q Network
UAV	Unmanned Aerial Vehicle
RL	Reinforcement Learning
